# Utility of hematological and inflammatory biomarkers in predicting recovery in critical Covid-19 patients: Our experience in the largest Covid-19 treating center in Lebanon

**DOI:** 10.1371/journal.pone.0271393

**Published:** 2022-07-13

**Authors:** Hassan Salame, Rashad Nawfal, Jad Kassem, Remy Mckey, Ali Kassem, Nayef AlKhalil, Mohamad Saleh, Ali H. Abdel Sater, Ali Ibrahim, Linda Abou-Abbas, Oussaima Eldbouni, Hoda Khatoun, Bassam Matar

**Affiliations:** 1 Department of Internal Medicine, Faculty of Medical Sciences, Lebanese University, Hadat, Lebanon; 2 Department of Surgery, Faculty of Medical Sciences, Lebanese University, Hadat, Lebanon; 3 Neuroscience Research Center, Faculty of Medical Sciences, Lebanese University, Hadat, Lebanon; 4 Department of Infectious Diseases, Rafic Hariri University Hospital, Beirut, Lebanon; 5 Department of Diagnostic Radiology, Saint-George Hospital, Beirut, Lebanon; 6 Department of Hematology and Oncology, Faculty of Medical Sciences, Lebanese University, Hadat, Lebanon; Stanford University School of Medicine, UNITED STATES

## Abstract

**Background:**

COVID-19 pandemic has led to a catastrophic shortage of ICU beds. This has resulted in the need to identify patients that can be discharged early before full clinical recovery. We designed this study to determine if in changes routine tests like CBCD and CRP can be a useful complement to clinical status when deciding to discharge patients from ICU.

**Methods:**

This retrospective study was conducted in Rafic Hariri University Hospital. Levels of biomarkers measured at admission (T1) and within 3 days of outcome (T2) were collected and ratios (T2/T1) were calculated. The Odds Ratios of association between the changes in these biomarkers and outcome were estimated. Multivariate analysis and AUC for the performance of these biomarkers were also conducted.

**Results:**

We found on multivariate analysis that reduction in counts of lymphocyte and platelets and elevation in counts of neutrophils and level of CRP (T2/T1 ratio > 1) are strongly associated with mortality with respective ORs estimated at 6.74, 3.26, 5.65 and 4.34 [p-values < 0.001]. AUCs were found to lie in a range of 0.68 to 0.81 indicating fair to good performance. Other factors found to impact survival were AKI, AF and ACS [p-values < 0.01]. In contrast to other studies, risk factors didn’t show an association with survival when adjusted for effects of complications and changes in biomarker levels.

**Conclusions:**

Our results confirm that inexpensive tests like lymphocyte count and CRP can be reliably used to follow COVID-19 patients in ICU and to support the decision to discharge patients.

## Introduction

Despite being around for several months, sars-cov2, and its disease coronavirus disease 2019 (COVID-19), are still making their way to headline news. Having been widely spread in a relatively short amount of time, it was inevitable to see new variants of this virus emerging and threatening the hope of recovery to our pre-pandemic life. It is now undebatable that this disease will accompany us in the coming years not only due to the mutations altering its genome but also due to the doubt still surrounding the longevity of the immunity induced by the vaccines protective against it [1].

Although, China was the epicenter of the disease in late 2019, it has spread rapidly to other parts of the world with the first wave peaking in Europe and North America in early 2020. Then, different sorts of restrictions were applied around the world and were successful to curb the spread of COVID-19 but only temporarily with resurge in cases after each attempt to ease these restrictions [2–4]. Alongside the unprecedented pressure put on health systems, the race to develop protective vaccines was fueled by the universal belief that this is our only hope to overcome this pandemic that has created unique challenges to modern society [5]. Fortunately, this global collaborative effort has finally succeeded in making these vaccines available to the public despite delays in vaccine rollout campaigns due to insufficient production capacities [6, 7]. Currently, the world is eyeing the end of this pandemic and preparing to keep the virus and its variants under control in the next years.

In Lebanon, we had an interesting epidemiological model of disease spread almost unique when compared to other countries in the region and the world. Early 2020, while other countries were counting cases in thousands, Lebanon was one of the few countries in the world that was reporting only dozens of cases daily. It wasn’t until late summer that cases in Lebanon started to rise rapidly breaking the threshold of 1000, 3000 and 5000 daily cases in mid-September, end of December and first week of January, respectively [8]. Yet, experts estimated that the real case toll is much more higher giving the lack of access to free testing among other factors. The consequences were devastating to the Lebanese health system that was hit at same time by a severe financial crisis that made resources necessary to save lives scant [9].

In a low-resource countries like Lebanon, this overwhelming stress on the health system led to a severe shortage of intensive care unit (ICU) beds [10]. This has compelled physicians in ICU to make every effort in order to free ICU beds including the use of inflammatory and non-inflammatory biomarkers like c-reactive protein (CRP), interleukin-6 (IL-6), d-dimers, creatine phosphokinase (CPK), lactate dehydrogenase (LDH) and ferritin to decide whether patients are stable enough to continue their recovery in non-ICU settings. As a result, medical laboratories have struggled to respond to the unprecedented demand on laboratory tests. Therefore, there was a need to determine what biomarkers are most accurate to follow in ICU and whether less costly tests like complete blood count and differential (CBCD) can perform similarly to fancier ones like IL-6.

We report in this paper the results of a retrospective study that evaluated the performance of different biomarkers in predicting mortality when followed in ICU settings. We also report the characteristics and outcomes of COVID-19 patients admitted to ICU in the largest COVID-19 treating center in Lebanon.

## Material and methods

### Study design and population

This retrospective study was conducted in Rafic Hariri University Hospital (RHUH) which is the largest COVID-19 care unit in Beirut. Patients included were those confirmed microbiologically to have COVID-19 and admitted to ICU either for critical disease or for severe disease with risk factors for deterioration between March 2020 and April 2021. Disease severity classification was based on clinical and radiologic criteria suggested by the World health organization [11]. Patients excluded are those who were admitted to treat another condition with only mild COVID-19 symptoms, discharged against medical advice, transferred to another hospital before clinical recovery or had incomplete medical records. Pregnant women and children were also not included in this study.

### Ethics approval and consent to participate

This study was approved by the institutional review board (IRB) of RHUH under the approval number 2021–0302. No contact of any type was made with patients and any information that can reveal patients’ identities was concealed; thus, the requirement for consent was waived by the IRB.

### Data collection

Medical records were accessed electronically and the data extracted was of the following types: demographics [age, gender, body mass index (BMI), smoking status], medical comorbidities [diabetes mellitus (DM), hypertension (HTN), cardiovascular disease (CVD), heart failure (HF), chronic kidney disease (CKD), chronic lung disease (CLD)], symptoms [fever, dyspnea, cough, sore throat, headache, fatigue, nausea/vomiting (NV), diarrhea], antibiotics, other treatments (steroids, anticoagulation, remdesivir, tocilizumab, convalescent plasma and vitamin D), results of cultures (respiratory tract, other body sites or fluids), complications [acute kidney injury (AKI), elevated liver enzymes (ELE), septic shock, atrial fibrillation (AF), acute coronary syndrome (ACS), stroke and deep vein thrombosis (DVT)] values of laboratory tests. Concerning the latter, two values of each test were collected: The value measured at admission (T1) and the last value available within 3 days of discharge or death (T2). For treatment with steroids, patients were divided into those who received standard dose for COVID-19 patients on high flow oxygen, that is 6 mg dexamethasone or equivalent [12], those who were treated with higher doses and those who received pulse therapy (500 mg to 1 g of solumedrol once daily for 3 days). In addition, patients were categorized into two groups depending on the highest dose of anticoagulation they received: prophylactic dose (40 mg of enoxaparin once daily or equivalent) or therapeutic dose (any dose higher than that used for prophylaxis). For treatment with antibiotics, patients who received a narrow-spectrum agent (ceftriaxone, azithromycin or levofloxacin) then a broad-spectrum agent (carbapenem family, colistin or piperacillin/tazobactam) were counted as if they had only received a broad-spectrum agent. Methicillin-resistant staphylococcus aureus (MRSA) coverage consisted of either vancomycin, teicoplanin or linezolid. The patterns for ELE are: hepatotoxic when only aspartate transaminase (AST) and/or alanine transaminase levels (ALT) were elevated, cholestatic when only gamma-glutamyl transpeptidase level (GGT) was increase and mixed when both patterns were present. AKI was defined following KDIGO (Kidney Disease Improving Global Outcomes) criteria [13]. Septic shock was defined as hypotension (MAP <65 mmHg) refractory to fluid resuscitation and necessitating norepinephrine administration. Sepsis-3 definition for septic shock was not adopted as we were not able to find lactate measurements for some patients which is a problem recognized by the task force that has established this definition, especially in developing countries like Lebanon [14]. The occurrence of other complications was determined based on clinical and biochemical evidence as well as progress notes available in medical files.

### Laboratory tests

COVID-19 diagnosis was made by detection of sars-cov2 nucleic acid on a nasopharyngeal swab using real time reverse transcriptase polymerase chain reaction (RT-PCR) assay performed on an Applied biosystems 7500 RT-PCR system (Thermo Fischer Scientific, Massachusetts, USA). CBCD [red blood cells (RBC), white blood cells (WBC), lymphocytes count, neutrophils count, monocytes count and platelets count) were measured with CELL-DYN Ruby Hematology analyzer (Abbott core laboratory systems, Illinois, USA). CRP, CPK, ALT, AST, GGT and d-dimers were measured on COBAS 6000 series chemistry analyzer (Roche diagnostics, Basel, Switzerland). IL-6, procalcitonin, ferritin and troponin on a Roche COBAS E601 electrochemiluminescence immunoassay analyzer.

### Statistical analysis

Data was analyzed using R software version 4.0.5 (R Foundation for Statistical computing). Death and invasive mechanical ventilation (IMV) were represented as counts and frequencies and length of time related to these outcomes were presented as median and interquartile range [25^th^– 75^th^]. All other data were represented in function of the survival status. Categorical variables were represented as frequencies and percentages. Continuous variables were represented as median and interquartile range. Normality test for the continuous variables was checked using the QQ plots and histograms. Chi-square and fisher’s exact tests were used to compare frequencies of categorical variables and Mann-Whitney U test to compare the medians of continuous variables. Ratios of values at T2 to those at T1 (ratios T2/T1) were categorized into two groups (cut-off = 1): Reduction (ratio ≤ 1) and elevation (ratio >1). Univariate logistic analysis was performed to estimate the odds ratios for association between change in laboratory tests (elevation vs reduction) and survival status. Only tests which were found to have statistically significant difference in ratios were included in this analysis. Receiver operating characteristic (ROC) curves and area under curve (AUC) were computed on R software based on univariate logistic analysis of association between ratios (T2/T1) as a continuous variable and survival status. AUC <0.7, 0.7–0.8, > 0.8 were deemed bad, fair and good respectively. Factors found to be associated with survival in univariate analysis were included in multivariate logistic regression. For association tests, we presented the OR, 95% confidence intervals and the p-value (significance level = 0.05).

## Results

### Outcomes of patients: Mortality rate, IMV and Length of hospitalization (LOH)

This study included 424 patients diagnosed with COVID-19 and admitted to ICU. We recorded a mortality rate of 49.1% (208 of 424) among enrolled participants. The median length of hospitalization (LOH) was 12 days [IQR 8–17]. Out of 424 patients, 10.6% died within the first 72 hours post-admission to ICU and median length of survival (LOS) was 4 [IQR 1–11] days. In addition, 138 (66.3%) of deceased patients received MV compared to 6 (2.8%) of alive patients (p-value < 0.001). Median length of time (LOT) before MV was longer in deceased patients 4.5 [IQR 1–11] compared to patients who survived 1 [IQR 0–3] (p-value = 0.019). Noting that IMV rate was 9.6% (41 of 424) within the first 24 hours of admission. These data are represented in Table 1.

**Table 1:** Outcomes of COVID-19 patients admitted to ICU.

		Total (N = 424)
**IMV**		144 (34.0%)
**Death**		208 (49.1%)
**LOT before MV (days)**	**Median [IQR]**	4.00 [1.00–11.00]
**LOS (days)**		11.00 [6.00–20.25]
**LOH (days)**		12.00 [8.00–17.00]

LOH = Length of hospitalization, LOS = length of survival, LOT = Length of time

### Demographic characteristics, symptoms and comorbidities

Table 2 represents demographic characteristics, symptoms and comorbidities of patients in function of the survival status. Among our participants, 270 (63.7%) were males, while 154 (36.3%) were females and there was no statistically significant difference between mortality rate and gender (p-value = 0.262). Median age of the study population reached 62 years [IQR 50.75–72.25] with a median age of 67 [IQR 56.8–77.3] for deceased patients that was significantly higher than that for alive patients (57 [IQR 46–68]) (p-value < 0.001). The leading underlying chronic condition in our population was HTN (59.2%), followed by DM (36.7%) and CVD (23.5%). Only 7.6% had HF, 9.5% had CKD, and 9.7% had CLD. Out of 424 patients, 22.5% were smokers, and median BMI was 28 kg /m^2^ [IQR 25.32–31.98] with no significant difference between survivors and non-survivors. Survival was associated negatively with HTN (p-value = 0.002), CVD (p-value = 0.004), HF (p-value = 0.001), and CKD (p-value = 0.005), where among the 208 deceased patients, 67% had HTN, 29.6% had CVD, 12.1% had HF, and 13.6% had CKD compared to alive patients (51.9%, 17.6%, 3.2% and 5.6% respectively). Out of 424 patients, COVID-19 symptoms were tracked at admission, noting fever (42.8%), dyspnea (92.8%), cough (64.4%), sore throat (3.6%), headache (3.3%), fatigue (38.8%), NV (6.7%) and diarrhea (9.3%). Furthermore, gastrointestinal symptoms rate was higher in alive patients (NV 9.3%; diarrhea 13%) compared to deceased patients (NV 4%; diarrhea 5.4%) (p-value = 0.030 and p-value = 0.008 respectively), while dyspnea was more frequently seen among patients who died (96.0% vs. 89.7% in patients who survived, p = 0.014) (Table 2).

**Table 2:** Demographics, medical comorbidities and symptoms.

		Survivors (N = 216)	Death (N = 208)	Total (N = 424)	p-value
**Demographics**					
**Gender**	Male	132 (61.1%)	138 (66.3%)	270 (63.7%)	0.262
	Female	84 (38.9%)	70 (33.7%)	154 (36.3%)	
**Age**	Median [IQR]	57 [46–68]	67 [56.8–77.3]	62 [50.75–72.25]	0.000
**Comorbidities**					
**DM**		78 (36.1%)	77 (37.4%)	155 (36.7%)	0.787
**HTN**		112 (51.9%)	138 (67.0%)	250 (59.2%)	0.002
**CVD**		38 (17.6%)	61 (29.6%)	99 (23.5%)	0.004
**HF**		7 (3.2%)	25 (12.1%)	32 (7.6%)	0.001
**CKD**		12 (5.6%)	28 (13.6%)	40 (9.5%)	0.005
**CLD**		20 (9.3%)	21 (10.1%)	41 (9.7%)	0.771
**Smoking**		47 (21.8%)	48 (23.3%)	95 (22.5%)	0.705
**BMI >30**		29 (33.7%)	36 (32.1%)	65 (32.8%)	0.815
**Symptoms**					
**Fever**		102 (47.2%)	77 (38.1%)	179 (42.8%)	0.060
**Dyspnea**		192 (89.7%)	192 (96.0%)	384 (92.8%)	0.014
**Cough**		146 (67.6%)	123 (60.9%)	269 (64.4%)	0.153
**Sore throat**		8 (3.7%)	7 (3.5%)	15 (3.6%)	0.896
**Headache**		9 (4.2%)	5 (2.5%)	14 (3.3%)	0.337
**Fatigue**		93 (43.1%)	69 (34.2%)	162 (38.8%)	0.062
**NV**		20 (9.3%)	8 (4.0%)	28 (6.7%)	0.030
**Diarrhea**		28 (13.0%)	11 (5.4%)	39 (9.3%)	0.008

DM = diabetes mellitus, HTN = hypertension, CVD = cardiovascular disease, HF = heart failure, CKD = chronic kidney disease, CLD = chronic lung disease, BMI = body mass index

### Treatments

Table 3 represents rate of bacterial growth on cultures, antibiotics and other treatments administered to patients in function of the survival status. Deep tracheal aspirate/sputum culture was positive in 37 patients (8.8%) distributed between 4 (1.9%) alive patients and 33 (16%) deceased patients (p-value<0.001) while cultures from other biologic matrices, mainly blood and urine, were positive in 67 patients (16%) with a significant association with death. Notably, out of those 104 patients, 42 patients (40%) had extensively drug resistant germs that were seen more frequently in non-survivors than in survivors (37 (18%) vs. 5 (2.3%), p-value < 0.001). Almost all patients in our cohort received an antibiotic with ceftriaxone being the most commonly employed antibiotic in non-critical patients followed by levofloxacin and azithromycin (47.1%, 22.4% and 15.6% respectively). However, in over half of our cohort, a broader spectrum antibiotic was initiated with 37% of patients being treated with carbapenem and 14.2% were treated with piperacillin/tazobactam. Moreover, 26.4% received a coverage for MRSA and 10.3% were treated with Colistin. For anticoagulant treatment, out of 424 patients, 78.9% received a therapeutic dose while 21.1% received prophylactic dose. Steroid treatment at standard dosing regimen was used in 34.9% of patients while higher dosing and pulse therapy were used, respectively, in 58.7% and 1.7% of patients. Other treatments included remdesivir (5.6%), tocilizumab (2.9%), convalescent plasma (3.4%) and vitamin D (42.2%). These treatments were not found to be associated with death except for remdesivir and convalescent plasma which seem to have a negative impact on survival.

**Table 3:** Rate of bacterial superimposed infections and treatments administered (LINE 240).

		Survivors (N = 216)	Non-survivors (N = 208)	Total	p-value
**Cultures**					
**Respiratory tract** ^1^		4 (1.9%)	33 (16.0%)	37 (8.8%)	0.000
**Other cultures** ^2^		18 (8.4%)	49 (23.8%)	67 (16.0%)	0.000
**Extensively drug resistant (XDR) germ**		5 (2.3%)	37 (18.0%)	42 (10.0%)	0.000
**Treatments**					
**Carbapenem**		39 (18.5%)	115 (56.1%)	154 (37.0%)	0.000
**MRSA coverage**		20 (9.5%)	90 (43.9%)	110 (26.4%)	0.000
**Colistin**		2 (0.9%)	41 (20.0%)	43 (10.3%)	0.000
**Piperacillin/tazobactam**		24 (11.4%)	35 (17.1%)	59 (14.2%)	0.096
**Ceftriaxone**		143 (67.8%)	53 (25.9%)	196 (47.1%)	0.000
**Levofloxacin**		72 (34.1%)	21 (10.2%)	93 (22.4%)	0.000
**Azithromycin**		39 (18.5%)	26 (12.7%)	65 (15.6%)	0.103
**Anticoagulation** ^3^	Prophylactic dose	50 (24.2%)	34 (17.8%)	84 (21.1%)	0.121
	Therapeutic dose	157 (75.8%)	157 (82.2%)	314 (78.9%)	
**Steroids** ^3^	No steroids	13 (6.2%)	6 (3.1%)	19 (4.7%)	0.088
	Standard dose	76 (36.4%)	65 (33.3%)	141 (34.9%)	
	High dose	119 (56.9%)	118 (60.5%)	237 (58.7%)	
	Pulse therapy	1 (0.5%)	6 (3.1%)	7 (1.7%)	
**Remdesivir**		6 (2.8%)	17 (8.6%)	23 (5.6%)	0.012
**Tocilizumab**		4 (1.9%)	8 (4.0%)	12 (2.9%)	0.247
**Convalescent plasma**		3 (1.4%)	11 (5.6%)	14 (3.4%)	0.028
**Vitamin D**		92 (42.6%)	87 (41.8%)	179 (42.2%)	0.873

^1^. Respiratory tract culture = deep tracheal aspirate and sputum cultures,

^2^. other cultures = blood, urine and wound cultures,

^3^. Refer to data collection section for definitions.

### Complications

The top four complications in the 424 patients were ELE (58.9%) (mixed pattern: 46.5%), AKI (48%) and septic shock (27.4%). AKI rate was higher in deceased patients (74.5%) compared to alive patients (22.6%) (p-value < 0.001) and septic shock rate was higher in death patients (54.5%) compared to alive patients (1.9%) (p-value < 0.001). In addition, less frequent complications, including ACS, AF and stroke, were more common in patients who died compared to participants who survived (p-value<0.05) (Table 4).

**Table 4:** Complications.

	Survivors (N = 216)	Non-survivors (N = 208)	Total	p-value
**AKI**	47 (22.6%)	149 (74.5%)	196 (48.0%)	0.000
**Elevated liver enzymes**	117 (55.5%)	128 (62.4%)	245 (58.9%)	0.148
ELE Hepatotoxic	16 (13.7%)	31 (24.2%)	47 (19.2%)	0.036
ELE Cholestatic	43 (36.8%)	41 (32.0%)	84 (34.3%)	0.437
ELE mixed pattern	58 (49.6%)	56 (43.8%)	114 (46.5%)	0.361
**Septic shock**	4 (1.9%)	108 (54.5%)	112 (27.4%)	0.000
**ACS**	4 (1.9%)	34 (17.2%)	38 (9.3%)	0.000
**AF**	7 (3.3%)	33 (16.7%)	40 (9.8%)	0.000
**Stroke**	0 (0.0%)	6 (3.0%)	6 (1.5%)	0.012
**DVT**	3 (1.4%)	2 (1.0%)	5 (1.2%)	1.000

AKI = acute kidney injury, ELE = elevated liver enzymes, AF = atrial fibrillation, ACS = acute coronary syndrome, DVT = deep vein thrombosis

### Laboratory tests in COVID-19 on admission to ICU

**Fig 1:**
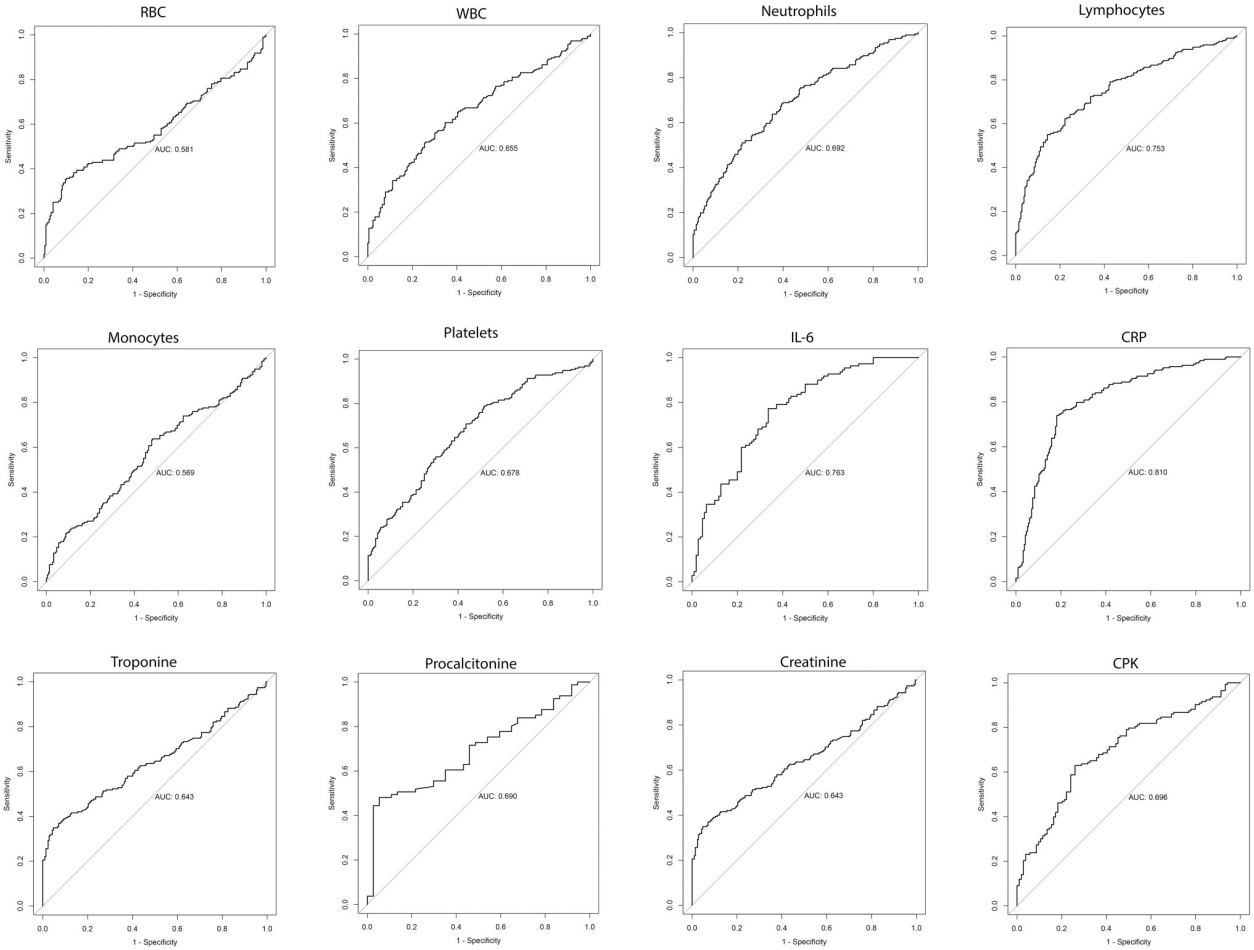
Comparison of the means of values of different laboratory tests at T1 and T2 between survivors and non-survivors.

The values of laboratory tests measured at admission (T1) and the ratios between the values measured at T2 and those measured at T1 are presented in Table 5. We also show in Fig 1 the changes in values of different laboratory tests between T1 and T2 and how the rates of these changes differ between survivors and non-survivors. At admission, mortality was associated with high white blood cells (p-value = 0.004), high neutrophils count (p-value = 0.001), low lymphocytes count (p-value = 0.010), low platelets count (p-value = 0.005), high AST level (p-value = 0.035), high creatinine level (p-value < 0.001), high LDH level (p-value < 0.001), high CPK level (p-value < 0.001), high d-dimers level (p-value = 0.002), high procalcitonin level (p-value < 0.001), high CRP level (p-value = 0.001), high IL-6 level (p-value < 0.001). Missing values at T1 were considerable (>10%) for LDH, d-dimers, procalcitonin and IL-6 which were not measured, respectively, in 42%, 39%, 34% and 12% out of 424 patients. To be noted that over the half of the survivors in our cohort had, at admission, abnormal values on neutrophils count, AST, LDH, d-dimers, CRP and Il-6 levels.

**Table 5:** Comparison of laboratory results between alive and death groups (LINE 268).

Laboratory test [normal range]		At Admission (T1)			Ratio (T2/T1)		
		Survivors	Non-survivors	P	Survivors	Non-survivors	P
**RBC [4.5–6.5 cells/μL]**	Median [IQR]	4.73 [4.3–5.1]	4.68 [4.1–5.2]	0.61	0.94 [0.9–1.0]	0.92 [0.8–1.00]	0.01
	Missing	0 (0.0%)	1 (0.5%)		0 (0.0%)	12 (5.8%)	
WBC [4–11 * 10^**3**^ **cells/μL]**	Median [IQR]	9.05 [6.4–12.7]	10.60 [7.79–14.90]	0.00	1.03 [0.8–1.4]	1.38 [1.0–2.0]	0.00
	Missing	0 (0.0%)	1 (0.5%)		0 (0.0%)	12 (5.8%)	
Neutrophils [1.6–7.15 * 10^**3**^ **cells/μL]**	Median [IQR]	7.71 [4.89–11.1]	9.14 [6.32–1.28]	0.00	0.98 [0.6–1.4]	1.49 [1.01–2.17]	0.00
	Missing	0 (0.0%)	1 (0.5%)		0 (0.0%)	12 (5.8%)	
Lymphocytes [1–4.95 * 10^**3**^ **cells/μL]**	Median [IQR]	0.96 [0.67–1.40]	0.81 [0.56–1.26]	0.01	1.22 [0.9–2.0]	0.68 [0.4–1.1]	0.00
	Missing	0 (0.0%)	1 (0.5%)		0 (0.0%)	12 (5.8%)	
**Platelets [150–400 10**^**3**^ **cells/μL]**	Median [IQR]	241.00 [181.0–311.0]	211.00 [157.0–281.8]	0.00	1.28 [0.9–1.6]	0.93 [0.6–1.3]	0.00
	Missing	0 (0.0%)	2 (1.0%)		0 (0.0%)	13 (6.3%)	
**Monocytes [0.08–0.8 10**^**3**^ **cells/μL]**	Median [IQR]	0.46 [0.31–0.70]	0.45 [0.32–0.67]	0.84	1.26 [0.8–1.8]	1.09 [0.7–1.6]	0.02
	Missing	1 (0.5%)	1 (0.5%)		1 (0.5%)	12 (5.8%)	
**AST [0–40 IU/L]**	Median [IQR]	43.00 [29.0–69.0]	49.00 [31.0–79.0]	0.04	0.67 [0.5–1.0]	0.84 [0.5–1.4]	0.08
	Missing	3 (1.4%)	3 (1.4%)		147 (68.1%)	109 (52.4%)	
**ALT [0–41 IU/L]**	Median [IQR]	32.00 [18.0–54.0]	31.00 [18.0–44.3]	0.41	0.97 [0.8–1.3]	1.12 [0.6–1.8]	0.27
	Missing	3 (1.4%)	4 (1.9%)		145 (67.1%)	108 (51.9%)	
**GGT [8–60 IU/L]**	Median [IQR]	57.00 [32.0–110.0]	56.00 [33.0–112.0]	0.93	1.08 [0.9–1.5]	1.16 [0.8–2.2]	0.42
	Missing	7 (3.2%)	4 (1.9%)		148 (68.5%)	114 (54.8%)	
**Creatinine [0.7–1.2 mg/dl]**	Median [IQR]	0.87 [0.73–1.12]	1.16 [0.87–1.87]	0.00	0.81 [0.68–0.95]	0.94 [0.73–1.53]	0.00
	Missing	4 (1.9%)	2 (1.0%)		4 (1.9%)	13 (6.3%)	
**LDH [135–225 IU/L]**	Median [IQR]	398.00 [312.0–531.0]	536.00 [430.0–703.0]	0.00	1.00 [0.7–1.1]	1.04 [0.7–1.6]	0.62
	Missing	98 (45.4%)	81 (38.9%)		201 (93.1%)	179 (86.1%)	
**CPK [20–200 μg/L]**	Median [IQR]	111.00 [59.0–311.0]	183.00 [87.0–416.0]	0.00	0.40 [0.2–0.9]	1.06 [0.5–2.3]	0.00
	Missing	5 (2.3%)	8 (3.8%)		112 (51.9%)	64 (30.8%)	
**d–dimers [0–0.5 g/L]**	Median [IQR]	0.85 [0.5–2.5]	1.57 [0.6–6.8]	0.00	1.63 [0.6–4.4]	2.73 [1.1–7.1]	0.1
	Missing	93 (43.1%)	72 (34.6%)		180 (83.3%)	144 (69.2%)	
**Troponin [0–0.014 ng/mL]**	Median [IQR]	0.01 [0.01–0.02]	0.03 [0.01–0.08]	0.00	0.88 [0.5–1.0]	1.39 [0.8–5.6]	0.00
	Missing	6 (2.8%)	4 (1.9%)		104 (48.1%)	44 (21.2%)	
**Procalcitonin [0–0.5 ng/mL]**	Median [IQR]	0.129 [0.1–0.3]	0.44 [0.2–1.2]	0.00	0.56 [0.3–1.0]	1.10 [0.50–3.7]	0.00
	Missing	81 (37.05%)	61 (29.3%)		179 (82.9%)	127 (61.1%)	
**CRP [0–5 mg/L]**	Median [IQR]	124 [64.6–189.0]	148 [97.8–233.0]	0.00	0.16 [0.1–0.3]	0.74 [0.4–1.3]	0.00
	Missing	0 (0.0%)	0 (0.0%)		1 (0.5%)	20 (9.6%)	
**Ferritin [30–400 ng/ml]**	Median [IQR]	701 [396–1250]	2070 [628–2000]	0.00	0.88 [0.6–1.6]	0.86 [0.6–1.4]	0.95
	Missing	90 (41.7%)	83 (39.9%)		195 (90.3%)	175 (84.1%)	
**IL–6 [0–7 pg/mL]**	Median [IQR]	42.50 [16.1–83.2]	83.0 [36.2–167.0]	0.00	0.17 [0.06–0.49]	0.66 [0.35–3.13]	0.00
	Missing	29 (13.4%)	20 (9.6%)		106 (49.1%)	98 (47.1%)	

RBC = red blood cells, WBC = white blood cells, AST = aspartate transaminase, ALT = alanine transaminase, GGT = gamma-glutamyl transpeptidase, LDH = lactate dehydrogenase, CPK = creatine phosphokinase, CRP = C-reactive protein, IL-6 = interleukin 6

Furthermore, we also found that the difference in ratios between survivors and non-survivors was significant for all laboratory tests except AST, ALT, GGT, LDH and d-dimers (Table 5). However, values at T2 for CPK, troponin, procalcitonin and IL-6, consequently their respective ratios, were not available in more than 10% of participants.

When ratios were converted into categories (elevation vs reduction), we found that survival was negatively associated with the elevation of WBC (p-value < 0.001; OR = 2.21 [95% CI 1.47–3.35]), neutrophils count (p-value < 0.001; OR = 3.36 [2.22–5.15]), creatinine (p-value < 0.001; OR = 3.505 [95% CI 2.225–5.521]) CPK (p-value < 0.001; OR = 3.30 [95% CI 1.91–5.83], troponin (p-value < 0.001; OR = 5.46 [95% CI 3.22–9.46]), procalcitonin (p = 0.003; OR = 3.90 [95% CI 1.65–10.01]), CRP (p-value < 0.001; OR = 7.21 [95% CI 4.10–13.40]) and IL–6 (p-value < 0.001; OR = 5.31 [95% CI 2.649–10.194]). In addition, our data showed that patients who had reduction in lymphocytes count (p-value < 0.001, OR = 4.95 [95% CI 3.27–7.58]) and platelets count (p-value < 0.001, OR = 2.70 [95% CI 1.81–4.05]) were more likely to die than those who had elevation in those counts (Table 6).

**Table 6:** Comparison of labs results between alive and death groups.

	Survivors N (%)	Non–survivors N (%)	Total N (%)	OR [95% CI]	p–value	AUC
**Elevation**						
**WBC**	116 (53.7%)	141 (67.8%)	257 (60.6%)	2.21 [1.47–3.35]	<0.001	0.66
**Neutrophils**	103 (47.7%)	147 (75.0%)	250 (60.7%)	3.36 [2.22–5.15]	<0.001	0.69
**Creatinine**	37 (17.5%)	83 (42.6%)	120 (29.5%)	3.505 [2.24–5.57]	<0.001	0.64
**CPK**	25 (24.0%)	73 (50.7%)	98 (39.5%)	3.30 [1.91–5.83]	<0.001	0.70
**Troponin**	27 (24.1%)	104 (63.4%)	131 (47.5%)	5.46 [3.22–9.46]	<0.001	0.73
**Procalcitonin**	8 (21.6%)	42 (51.2%)	50 (42.0%)	3.90 [1.65–10.01]	0.003	0.69
**CRP**	16 (7.4%)	69 (36.7%)	85 (21.1%)	7.21 [4.10–13.40]	<0.001	0.81
**IL–6**	14 (12.6%)	48 (42.9%)	62 (27.8%)	5.31 [2.76–10.75]	<0.001	0.76
**Reduction**						
**RBC**	59 (27.3%)	51 (26.0%)	110 (27.7%)	1.07 [0.69–1.66]	0.767	0.58
**Lymphocytes**	141 (65.3%)	54 (26.0%)	195 (46%)	4.95 [3.27–7.58]	<0.001	0.75
**Monocytes**	140 (65.1%)	111(56.6%)	251 (61.1%)	1.43 [0.96–2.13]	0.079	0.57
**Platelets**	146 (67.6%)	85 (43.6%)	231 (56.2%)	2.70 [1.81–4.05]	<0.001	0.68

WBC = white blood cells, CPK = creatine phosphokinase, CRP = C-reactive protein, IL-6 = interleukin 6, RBC = red blood cells

The performance of the changes in these laboratory tests in terms of correctly classifying patients by their survival status was evaluated with ROC curve analysis (Fig 1). It showed that the change in CRP level had a good prognostic accuracy (AUC>0.8) while changes in CPK, troponin, IL–6 levels and lymphocytes count had only a fair accuracy (AUC = 0.7–0.8). Remaining tests had a bad performance on this analysis (AUC = 0.6–0.7).

### Multivariate logistic regression: Factors affecting survival

The model included all factors (Table 7) which had statistically significant association with mortality in the univariate settings and had missing values less than 10%. It showed that mortality was associated with the presence of AF (p-value = 0.002; OR = 5.77 [2.02–19.15]), ACS (p-value = 0.002; OR = 8.10 [2.44–33.95]), AKI (p-value < 0.001; OR = 9.37 [5.03–18.22]), an elevation (ratioT2/T1>1) in neutrophils counts (p-value < 0.001; OR = 5.65 [2.92–11.49]) CRP level (p-value <0.001; OR = 4.34 [2.04–9.68]). In addition, as in univariate analysis, mortality remained significantly associated with reduction (ratio T2/T1 ≤1) in lymphocytes count (p-value<0.001; OR = 6.74 [3.64–12.99]) and platelets count (p<0.001; OR = 3.26 [1.75–6.25]).

**Table 7:** Multivariate logistic regression analysis for the factors affecting the survival.

	Adjusted OR [95% CI]	p–value
**Age**	1.02 [0.99–1.04]	0.201
**CKD**	0.96 [0.34–2.72]	0.93
**HF**	2.77 [0.85–10.00]	0.101
**HTN**	0.52 [0.24–1.08]	0.083
**CVD**	0.98 [0.43–2.22]	0.962
**AF**	5.77 [2.02–19.15]	0.002
**ACS**	8.10 [2.44–33.95]	0.002
**AKI**	9.37 [5.03–18.22]	0.000
**CRP [Elevation]**	4.34 [2.04–9.68]	0.000
**Lymphocytes [Reduction]**	6.74 [3.64–12.99]	0.000
**Neutrophils [Elevation]**	5.65 [2.92–11.49]	0.000
**Platelets [Reduction]**	3.26 [1.75–6.25]	0.000

CKD = chronic kidney disease, HF = heart failure, HTN = hypertension, CVD = cardiovascular disease, AF = atrial fibrillation, ACS = acute coronary syndrome, AKI = acute kidney injury, CRP = c-reactive protein.

## Discussion

Among COVID–19 patients admitted to ICU, we found that 34% received IMV after a median time of 4 days and that 49.1% died after a median time of 11 days after admission. Those who survived had to stay in the hospital for a median time of 12 days before recovery. Similar results for mortality rate were reported in other ICU case series in Lombardy, Italy [15] and Seattle, USA [16] where, respectively, 48.3% and 50% of critically ill patients didn’t survive the disease. However, IMV rate in our study was much lower than those reported by these two studies (87.3% and 75% respectively). This finding can be explained by the high variability in the availability of human and logistic resources at each center which could have tightened the indications for endotracheal intubation. In fact, in our cohort, 78 out 424 (18.4%) patients had Do–not–resuscitate (DNR) orders instituted due to end–stage disease or old age with multiple comorbidities (median age of patients who had DNR orders was 79 years). This highlights the disastrous effects that the pandemic had on the health system and that led medical centers globally to pursue the agonizing approach of prioritizing the lives of patients based on their medical fitness before COVID–19 [17, 18].

Regarding prognostic factors for survival, our results are consistent for the most part with those reported in a systematic review that analyzed 207 studies [19]. On univariate analysis, we found that age, HTN, CKD, cardiovascular disease and heart failure are significant predictors of mortality. However, male sex, DM, CLD, smoking and obesity didn’t differ between survival groups in our study while they were found to provide valuable prognostic information with moderate to high certainty in the previously cited systematic review.

Dyspnea was the most commonly reported symptom at presentation. This was an expected finding given that respiratory involvement was the main indication for admission to ICU in our hospital. While we observed that dyspnea was more frequently reported by non–survivors and gastrointestinal symptoms was more frequently seen in survivors, symptoms were not found to be reliable predictors of mortality in other studies including a systematic review [19].

We presented in Table 3 the data on rates of superimposed bacterial infections and treatments administered. These are of exploratory type and aim to shed light on areas of clinical practice in treating COVID–19 patients that warrant further research. In our cohort, almost all patients were started empirically on antibiotics at ICU admission specifically ceftriaxone (47.1% of patients) alone or combined with either azithromycin (15.6%) or levofloxacin (22.4%). Broad spectrum antibiotics were initiated in patients who deteriorated clinically with carbapenems, MRSA coverage, piperacillin/tazobactam and colistin being used in 37%, 26.4%, 14.2% and 10.3% respectively. These rates of antibiotics use don’t reflect the rates of bacteria grown on cultures with the exception of cultures yielding extensively drug resistant germs (8.26%) that seem to account for most of the cases in whom colistin was administered (10.3%). Indeed, only 82 out of 424 patients (19.3%) had positive culture in at least one biological matrix and 37 patients (8.8%) had positive culture on respiratory tract secretions. The latter corresponds mostly (89.2%) to cultures from deep tracheal aspirates which were positive in 33 patients out of 144 patients who received IMV (23%). These results are comparable to those reported in a similarly designed study conducted in China which noted that 13.5% of patients had hospital acquired infections and that 94% of patients received antibiotics [20]. We also presented the association between the antibiotic used and mortality, but this was only to indicate that non–survivors have had been more frequently treated with broad spectrum antibiotics than survivors. In addition, we found that majority of patients received anticoagulation at a therapeutic dose (78.9%) while high dose steroid (higher than 6 mg dexamethasone) was used in 58.7% of patients. Therapeutic dosing of anticoagulation was driven by the emerging reports, at the time, indicating high risk of thrombotic complications in critically ill patients and the interim guidance by different medical societies including the European society of cardiology recommending therapeutic strategy in select patients that meet certain criteria in ICU and non–ICU settings [21]. Similarly, the reports on proinflammatory cytokine storm and its pulmonary and multisystemic effects, have supported the trial of higher dose steroids to downregulate this maladaptive immune response [22, 23]. On survival analysis, we were not able to detect any association with mortality. In fact, we found that among the 237 patients who received a high dose steroid and the 314 patients who received a therapeutic anticoagulation, nearly half of each group didn’t survive (49.8% for high dose steroids and 50% for therapeutic anticoagulation), which is consistent with the overall mortality rate (49.1%). However, these results should be interpreted carefully as, similarly to the selection bias in the spectrum of antibiotics used, the choice of the regimen (prophylactic vs therapeutic and standard vs higher dose) was based on the clinical status of the patient. Other treatments were less frequently used except for vitamin D which was administered in 42.2% of patients. Although, 74% and 79% of patients who, respectively, received remdesivir and convalescent plasma died of COVID–19, no final conclusions can be drawn as no matched groups were available.

Moreover, our results have also shown that liver injury is the most common complication in COVID–19 patients admitted to ICU. Different patterns of involvement were encountered with the mixed pattern being the most common overall. While hepatotoxic injury was found to be associated with death, septic shock and drug-related injuries are possible confounders that cannot be excluded. AKI and septic shock were also frequent complications that negatively impacted survival with only 24% and 3.6%, respectively, of patients have survived after developing these two complications. Early studies in Wuhan have noted that AKI occurred in 3.2% of hospitalized patients with COVID–19 [24], while later studies indicate that this is much more frequent complication occurring at a rate of 29% in ICU patients in Wuhan and 31% in critically ill adults in New York city. Furthermore, clinically relevant thrombotic complications were identified in 49 patients (11.6%). These are lower than rates in the study of Klok et al. where 31% of critically ill patients developed thrombotic events. An important distinguishing fact is that 9.2% of patients in that study were on therapeutic anticoagulation at admission while in our study 78.9% received therapeutic regimen [25].

Many studies have already reported an association between the values of different inflammatory and non–inflammatory biomarkers and prognosis. The majority of those studies have collected the values of those tests at admission. They have mainly compared the prognosis, between patients who had abnormal values on a particular laboratory test to those who had values within normal range of that test [26]. Therefore, those studies were not applicable to ICU settings where the vast majority of patients had abnormal values, relatively to normal range, on laboratory tests at admission (refer to section on Results). Furthermore, levels of biomarkers at admission are not expected to change management because patients tend to seek healthcare at variable points over the course of their disease and consequently it’s unknown whether values at admission represent peak or trough levels. In this context, we designed this study in order to inform physicians about the best laboratory tests, in terms of predictive power for survival, to follow in ICU patients and to support them in the decision to discharge a patient from ICU by providing a complement to clinical status.

To achieve this goal, we reported the ratios for the change in biomarkers levels between admission and the immediate period before reaching outcome (death vs discharge). We found that depending on their survival status, patients had significantly different trends in their counts of RBC, WBC, neutrophils, lymphocytes, monocytes and platelets and their levels of creatinine, troponin, CPK, procalcitonin, CRP and IL-6. Notably, d–dimers levels increased in both survivors (63% increase) and non–survivors (173% increase) but the difference was not found to be statistically significant different. To make results more useful in clinical settings, we compared the outcomes of patients who had elevation in their biomarkers levels to those who had reduction. We found that reduction in lymphocytes counts and elevation of CRP, IL–6, CPK and troponin levels are significantly associated with death. In addition, ROC curve analysis has demonstrated that these changes have fair to good predicting performance with AUC ranging from 0.7 to 0.81.

In the final multivariate model, we included laboratory tests who had less than 10% of missing values. Also, as mortality depends on multiple factors and not limited to changes in laboratory tests, demographic and clinical characteristics were also included in the final model. We also added significant complications to the multivariate model because we think they had a major impact on mortality, thus we wanted to adjust the effects of other variables to the effects of complications. However, septic shock and stroke were excluded due to almost perfect correlation with death. The final model revealed that patients who had elevation of CRP level and neutrophils count and those had reduction in lymphocytes and platelets counts were more likely to die than those who had the opposite changes. These findings are very similar to those reported by Wang et al. who analyzed the effects of change in biomarkers (troponin, d–dimers, CRP, IL–6, procalcitonin, lymphocytes and neutrophils) between values at admission and peak levels (increase vs decrease) and severity of disease. However, their cohort comprised only 77 patients and they didn’t perform multivariate analysis to adjust for clinical and demographic characteristics [27]. Furthermore, we found that the effects of predisposing conditions were no more statistically significant when adjusted for post-admission factors, that are complications and changes in biomarkers levels. This is in contrast to other studies which reported significant association between mortality and clinical characteristics when adjusted only to biomarkers levels at admission [28–31].

## Conclusion

Our results confirm that the changes in counts of neutrophils, lymphocytes and platelets measured on complete blood count and differential (CBCD) and CRP level are useful in predicting death from COVID–19 in ICU. While increases in levels of IL–6, CPK and troponin were also found to have a strong association with death on univariate analysis, we were not able to compare them, on multivariate analysis, to CBCD and CRP due to missing values. Nevertheless, given the low cost and wide availability of CBCD and CRP and their good predicting accuracy, we believe that provided evidence is sufficient to allow us suggesting to use these two tests rather than more costly tests, like IL–6, when deciding to discharge patient from ICU. In addition, our results suggest that it is of utmost importance to monitor the occurrence of complications in patients admitted to ICU and to manage early and aggressively those complications.

## Limitations

The choice of timepoint 2 as the last value in a range of 3 days preceding the occurrence of outcome (death or discharge) could raise some questions on the consistency of values measured at T2 when comparing one patient to another. We acknowledge that it would be desirable to fix T2 at a specific day after admission. However, this was not possible for the following reasons: the high variability of the length of time before reaching the outcome, the lack of standardization on the timing of measuring biomarkers and the necessity to choose a timepoint representative of the pathophysiologic changes near the time where the patient was stable enough to be discharged or the time of his death. These issues are inherent to the retrospective design of the study and we were not able to overcome them. Nevertheless, by tracking the change between levels at admission and levels in the immediate period before reaching the outcome, we were able to detect the trend in these biomarkers which was the aim of this study. Another limitation was the lack of sufficient data at the timepoint 2 for some biomarkers which is also secondary to the retrospective design of the study.

## Supporting information

S1 File Dataset. (XLSX)

S2 File (XLSX)

## Abbreviations

ACS: acute coronary syndrome
AF: atrial fibrillation
AKI: acute kidney injury
ALT: alanine transaminase
AST: aspartate transaminase
AUC: area under curve
BMI: body mass index
CBCD: complete blood count and differential
CKD: chronic kidney disease
CLD: chronic lung disease
COVID-19: coronavirus disease 2019
CPK: creatine phosphokinase
CRP: c-reactive protein
CVD: cardiovascular disease
DM: diabetes mellitus
DVT: deep vein thrombosis
ELE: elevated liver enzymes
GGT: gamma-glutamyl transpeptidase
HF: heart failure
HTN: hypertension
ICU: intensive care unit
IL-6: interleukin 6
IMV: invasive mechanical ventilation
IRB: institutional review board
KDIGO: kidney disease improving global outcomes
LDH: lactate dehydrogenase
LOH: Length of hospitalization
LOS: length of survival
LOT: Length of time
MRSA: methicillin-resistant staphylococcus aureus
NV: nausea/vomiting
RBC: red blood cells
RHUH: rafic hariri university hospital
ROC: receiver operating characteristic
RT-PCR: real time reverse transcriptase polymerase chain reaction
WBC: white blood cells
XDR: extensively drug resistant
